# The Bidirectional Relation Between Counterfactual Thinking and Closeness, Controllability, and Exceptionality

**DOI:** 10.3389/fpsyg.2022.732870

**Published:** 2022-03-09

**Authors:** Yibo Xie, Sarah R. Beck

**Affiliations:** ^1^Management School, Hainan University, Haikou, China; ^2^School of Psychology, University of Birmingham, Birmingham, United Kingdom

**Keywords:** counterfactual thinking, closeness, controllability, exceptionality, inference

## Abstract

In four experiments, we explored the inferences people make when they learn that counterfactual thinking has occurred. Experiment 1 (*N* = 40) showed that knowing that a protagonist had engaged in counterfactual thinking (compared to no counterfactual thinking) resulted in participants inferring that the past event was closer in time to the protagonist, but there was no difference in inferring how close the past event was between knowing that a protagonist made many or a single counterfactual statement(s). Experiment 2 (*N* = 80) confirmed that participants were not affected by the number of counterfactual statements they read when inferring temporal closeness. Experiment 3 (*N* = 49) demonstrated that participants who learned that a protagonist had engaged in counterfactual thinking were more likely to infer that the protagonist experienced the controllable event. Experiment 4 (*N* = 120) indicated that participants who learned that a protagonist had engaged in counterfactual thinking were more likely to infer that the protagonist experienced the exceptional event. We concluded that the existence (but not the number) of counterfactual thoughts can lead people to infer that events were close, exceptional, and controllable, which suggests that the relations between closeness/controllability/exceptionality and counterfactual thinking are bidirectional. These results showed that as well as making inferences based on facts about the real world, people also make inferences about the real world based on hypothetical worlds.

## Introduction

Counterfactual thinking is the mental simulation of events that could have occurred in the past but did not (Kahneman and Tversky, 1981; Decety and Ingvar, 1990). To think counterfactually one must ignore events that have occurred and represent the alternative that might have occurred instead (Roese, 1997). Counterfactual thinking often takes the form of counterfactual conditionals “if…, then…” (Stalnaker, 1999), for example, “If you had not bought the blue dress, then you would have bought the red one.” Research has mapped out the events that prompt people to engage in counterfactual thinking (e.g., Meyers-Levy and Maheswaran, 1992) and also the function and interpretation of counterfactual thoughts or statements (e.g., Epstude and Roese, 2008). Here, we focus on the latter and explore the inferences people make once they learn that counterfactual thinking has occurred. Specifically, we explore whether people can make closeness, controllability, and exceptionality inferences when they learn that counterfactual thinking has occurred.

A range of theoretical and empirical research has examined the function and interpretation of counterfactuals. In terms of function, a well-established theory is that counterfactuals allow us to make better decisions for the future by reflecting on how things could have been better. This can be the result of considering specific alternative actions or by impacting our motivation to repeat the same actions or not in the future (Epstude and Roese, 2008; Smallman and Summerville, 2018). Hammell and Chan (2016) provided evidence that considering counterfactuals resulted in improved performance on two physical tasks. Other functions of counterfactuals include supporting moral judgments (Byrne, 2016) and underpinning a sense that our life events are meaningful (McCrea, 2008).

This work on the function of counterfactuals relies on a fundamental interpretation that people make when they interpret a counterfactual statement, which is that the counterfactual describes a possibility which is false. From the statement in the opening paragraph we can infer that the individual did not buy the red dress. Substantial research has tested these inferences and generated theoretical frameworks to account for how possibilities with different epistemic status are represented. Prominent here is mental models theory which sets out to account for the different logical possibilities that are generated when considering a conditional, including counterfactuals (e.g., Byrne and Johnson-Laird, 2020).

Santamaría et al. (2005) primed participants with counterfactual or indicative conditional statements, e.g., “If there had been roses, then there would have been lilies” or “If there were roses, then there were lilies” and recorded how long they took to read subsequent conjunctions e.g., “There were roses and there were lilies.” When participants read the counterfactual conditional they were quicker to read a negative conjunction “There were no roses and there were no lilies” than when they had been primed by an indicative conditional. However, reading times for positive conjunctions “There were roses and there were lilies” were equally fast for both priming conditions. This was interpreted as showing that when people read a counterfactual conditional they hold in mind two possibilities [p and q (the counterfactual) and not p and not q (inferred reality)]. In contrast, on reading an indicative conditional, people tend to represent only one possibility (p and q). Espino and Byrne (2020) argued that people not only represent the counterfactual and real world, but that they also track the epistemic status of these possibilities; they expect stories about reality to continue to focus on reality, but stories including counterfactuals prime them to hold in mind both reality and the counterfactual.

As well as inferring that the counterfactual is false, other studies have examined people’s understanding of counterfactual statements. Researchers have mainly used sentence probe tasks (e.g., De Vega et al., 2007; De Vega and Urrutia, 2012) or measured participants’ reading times to explore what people understand when reading counterfactuals (e.g., Santamaría et al., 2005; Ferguson et al., 2008; Ferguson and Sanford, 2008; Ferguson, 2012; Ferguson and Jayes, 2018). For example, in Ferguson (2012), participants read a story starting with either a factual sentence (e.g., “Because Joanne had remembered her umbrella, she had avoided the rain.”) or a counterfactual sentence (e.g., “If Joanne had remembered her umbrella, she would have avoided the rain.”). Participants then read sentences that were consistent or inconsistent with the starting sentence. This experiment had three conditions: factual-consistent, counterfactual-consistent, and counterfactual-inconsistent. For example, in the counterfactual-consistent condition participants read “Joanne’s hair was wet” after reading the counterfactual sentence “If Joanne had remembered her umbrella, she would have avoided the rain”; and in the counterfactual-inconsistent condition participants read “Joanne’s hair was dry” after reading the counterfactual sentence. Participants had shorter reading times when reading the factual-consistent story than the counterfactual-consistent or counterfactual-inconsistent story and there was no difference in reading time between reading the counterfactual-consistent story and counterfactual-inconsistent story. It is more difficult for people to process counterfactual information than factual information reflecting that readers of counterfactual information need to take into account both counterfactual and factual information.

A further experiment (Ferguson and Sanford, 2008) showed that people make inferences that are consistent within the counterfactual world they are considering. For example, participants read a counterfactual statement (“If cats were vegetarians…”) followed by a continuation that was either consistent with the counterfactual world (but not reality) e.g., “Families could feed their cat a bowl of carrots…” or that was inconsistent with the counterfactual world (but was consistent with reality) e.g., “Families could feed their cat a bowl of fish.” The first pass reading times for these sentences showed that people were slower to read information that was inconsistent with their real world knowledge (carrots). However, the inconsistency was quickly ‘neutralized’ and participants were quicker to read the continuations that were consistent with the world they were set in, regardless of their absolute relation to reality.

The work we have described showed that people represent different alternative possibilities when understanding counterfactual information compared to factual information, and that they generate inferences consistent with the counterfactual world. However, one area yet to be explored is whether some specific inferences might arise from counterfactuals that are biased in the same way that the generation of counterfactual thinking is biased. This moves beyond the logical inferences that have been studied to date (the truth or falsity of a statement or the consistency of claims). We know that determinants exist that influence the tendency to engage in counterfactual thinking. Specifically, the tendency to engage in counterfactual thinking is more likely when there are particular determinants: these include *closeness* (e.g., Meyers-Levy and Maheswaran, 1992; Medvec et al., 1995; Medvec and Savitsky, 1997), *controllability* (e.g., Markman et al., 1995; Wrosch and Heckhausen, 2002), and *exceptionality* (e.g., Kahneman and Tversky, 1981; Gavanski and Wells, 1989). Our aim was to test whether the relation between these determinants and counterfactuals held only for generation, or whether the effect was seen in reverse when starting with a counterfactual statement. Thus, we now summarize evidence on these determinants and their influence on counterfactual thinking.

Closeness refers to the gap between the actual outcome of an event and the expected outcome that might have occurred. Closeness can refer to temporal closeness (e.g., missing a flight by just 5 min rather than 1 h), spatial closeness (e.g., being robbed when only 100 m rather than 2 km from one’s home) and numerical closeness (e.g., holding ticket number 99 in a lottery when number 100 wins the prize). For example, previous experiments (e.g., Meyers-Levy and Maheswaran, 1992; Roese and Olson, 1996 experiment 3) showed that temporal closeness to the expected outcome was more likely to lead to counterfactual thinking than temporal distance. Meyers-Levy and Maheswaran (1992) asked participants to read a story describing an apartment fire that occurred 3 days (i.e., temporal closeness) or 6 months (i.e., temporal distance) after the owner had forgotten to buy insurance. The results suggested that, compared with the participants who read the story about the fire 6 months later, participants who read the material about the fire 3 days later were more likely to attribute counterfactual thinking to the story protagonist. In other experiments (Roese and Olson, 1996), a story described a frustrated protagonist who failed to catch a plane by only 5 min (i.e., temporal closeness) or 1 h (i.e., temporal distance). Critically, the airport staff informed the protagonist that the plane had taken off at the scheduled departure time (i.e., 1 h before) or just 5 min before the protagonist arrived at the airport, so in each version the protagonist’s efforts to get to the airport on time were the same. The participants were asked to write down their thoughts about this story. Participants were more likely to generate counterfactual thoughts for the protagonist who missed the plane by only 5 min, i.e., was closer to catching it. Roese (1997) argued that the functional nature of counterfactual thinking means that we pay attention to events that almost happened, because these are ones we are likely to benefit from attempting to change in the future. However, it is important to note that Gilbert et al. (2004) suggested that while the effect of closeness is seen when participants imagine hypothetical scenarios, the impact is not so strong (or does not exist) when people actually experience the events.

As for controllability, many researchers (e.g., Girotto et al., 1991) suggest that people’s counterfactual thinking is more likely to focus on controllable rather than uncontrollable events. For example, Girotto et al. (1991) asked participants to generate counterfactual thoughts for one protagonist who was delayed by both controllable events (e.g., having a drink) and uncontrollable events (e.g., road blocked by a flock of sheep). On reaching home he found his wife had just died of a heart attack. Researchers found that participants’ counterfactual thoughts mainly focused on the protagonist’s controllable action (e.g., “he could have come home earlier and saved his wife if he did not have a drink”). Mandel and Lehman (1996) asked participants to read a story about a car accident and generate counterfactual thoughts for the protagonist. Results showed that participants’ counterfactual thoughts mainly focused on the controllable actions that protagonist did (e.g., “he should have chosen another route”). According to the functional theory (Epstude and Roese, 2008) counterfactual thinking focuses on controllable rather than uncontrollable events, because people can only change controllable events in the future.

Kahneman and Tversky (1981) suggested that people are more likely to think counterfactually about exceptional events than unexceptional events, demonstrating that exceptionality leads to counterfactual thinking. Researchers used two versions of stories both about a protagonist who suffered a car accident on the way home. One version of the story was that the protagonist left his office at an exceptional time, and stuck to his usual route. In the other version of the story the protagonist left his office at the usual time, but chose an exceptional route to go home. Participants thought counterfactually about how this protagonist could have been able to avoid this car accident. Results showed that participants’ counterfactual thoughts were more likely to focus on the exceptional than normal time or route (e.g., “If only he chose another way…”). Gavanski and Wells (1989) asked participants to read a story about a student taking an exam. Participants read the story that either had an exceptional outcome (i.e., the outstanding student did not pass the exam) or an unexceptional outcome (i.e., the outstanding student passed the exam). The story also included some exceptional (e.g., mother was suddenly sick) and unexceptional events (e.g., the student was normally nervous before the exam). A tendency to focus counterfactual thinking on the exceptional events was seen, but only when the outcome itself was also exceptional.

Intriguing research by Teigen (1998, 2005) shows a link between closeness and estimates of likelihood in a scenario that lent itself to counterfactual thinking. In one example two people were walking together down a path and one was hit by an icicle. Counterintuitively, with hindsight people often estimated the likelihood of the person who was not hit as higher than the one who was hit. Teigen explains this as the result of comparisons with a close counterfactual. For the person who was unharmed there is a compelling close counterfactual that they almost got hit. This influences the judgment of likelihood. This finding supports the claim that there is a strong relationship between the determinants of closeness, controllability, and exceptionality, and that people draw inferences based on counterfactuals. We will investigate this bidirectional relationship systematically and a simple and direct way in our studies.

If closeness, controllability, and exceptionality to the expected outcome can lead to counterfactual thinking, the reverse pattern might be also true. This question about what inferences people draw is important, because people not only engage in counterfactual thinking themselves, they also hear other people express counterfactual thoughts and need to interpret them. Interpreting others’ counterfactual statements allows people to make inferences about the original event. Thus, it is possible that we could influence participants’ estimates of the closeness, controllability, and exceptionality by manipulating participants’ exposure to counterfactuals.

The most prominent theory of counterfactual thinking is the functional theory (Epstude and Roese, 2008) which holds that counterfactual thinking underpins intentions to change behavior in the future, and consequently an actual change in future behavior. This is described as a preparatory function. Non-preparatory functions have also been proposed: counterfactual thinking can influence self esteem (McCrea, 2008) and can underpin a sense of meaningfulness about one’s life (Kray et al., 2010). In this paper we explore another non-preparatory function of counterfactuals, that of conveying information.

Hence, in four experiments we explored whether knowledge of another’s counterfactual thinking (*via* explicit statements) leads people to make inferences about closeness, controllability, and exceptionality: the reverse of the established effects that closeness, controllability, and exceptionality lead people to engage in counterfactual thought (or infer that others would engage in counterfactual thought). Our main question was whether there would be differences in inferences based on whether characters made statements about counterfactuals or just about reality, based on the previous literature on the determinants that prompt counterfactual thinking. We also included a second exploratory question: whether the extent of the thinking described (many versus single counterfactuals) would affect the likelihood of drawing such an inference. We speculated that including many statements would make the counterfactual thinking more salient and increase the likelihood of participants drawing inferences.

Specifically, in Experiment 1, based on previous closeness experiments (i.e., Meyers-Levy and Maheswaran, 1992; Roese and Olson, 1996 experiment 3), we tested the possibility that there may be differences in inferences about closeness based on whether characters made statements about counterfactuals or just about reality. In Experiment 2, we confirmed whether the extent of the thinking described (many versus single counterfactuals) affected the likelihood of drawing inference about closeness. In Experiment 3, based on Girotto et al. (1991) who demonstrated that controllability can lead people to think counterfactually, we hypothesized that the existence of counterfactual thinking would lead participants to infer that the event was controllable. Finally, we based Experiment 4 on Kahneman and Tversky (1981) who demonstrated that exceptional events can lead participant to think counterfactually; we hypothesized that the existence of counterfactual thinking would lead participants to infer that event was exceptional. Overall, our goal was to explore that whether there is a bidirectional relation between counterfactual thinking and counterfactual determinants.

## Experiment 1: Closeness

In Experiment 1, we explored the possible bidirectional relation between closeness and counterfactual thinking, by studying whether reading counterfactuals can lead to inferring closeness. Specifically, we tested whether there may be differences in inferring closeness between reading counterfactual and reality statements. We had two hypotheses in Experiment 1. Firstly we hypothesized that knowing that a protagonist made a counterfactual statement(s) would result in participants inferring that the past event was closer in time compared to a protagonist who made statement about reality. Secondly we hypothesized that knowing that a protagonist made many counterfactual statements will result in participants inferring that the past event was closer in time compared to a protagonist who made single counterfactual.

### Method

#### Participants

Forty-two psychology students (36 women and 6 men) from a university in a city in the United Kingdom participated in Experiment 1 to gain course credits. Participants in all experiments reported in this paper gave consent and all experiments were approved by the STEM Ethics Review Committee of [redacted for anonymous review]. No individual participated in more than one experiment reported in this paper. Participants across our four experiments were predominantly female (78.8–91.3%), reflecting the student cohort from which they were recruited.

#### Design and Materials

There were two conditions in Experiment 1: a many counterfactuals condition who read about a character generating several counterfactual statements about the event and a single counterfactual condition who read about a character generating just one counterfactual statement about the event. Participants in each condition also read a second version of the story with a character who made no counterfactual statement. Instead, this character made a statement that described reality. Participants in each condition read a pair of plane stories (counterfactual and reality) and a pair of fire stories (counterfactual and reality). Both stories had the same level of counterfactual statements, i.e., they either read single counterfactual versions of both plane and fire stories, or they read many counterfactual versions of both stories.

The plane story was adapted from Roese and Olson (1996, experiment 3). In the original, airport staff informed the protagonist that the plane had taken off at 1 h or 5 min before the protagonist arrived at the airport. We removed this information about timing and added the protagonist’s own comment on the events at the end of the story containing either many counterfactuals (many counterfactuals version), a single counterfactual (single counterfactual version), or a reality statement (reality version). The second story about an apartment fire was adapted from Meyers-Levy and Maheswaran (1992, experiment 2). In the original, the protagonist’s apartment suffered a fire 3 days (or 6 months) after he forgot to send the fire insurance application. This information was removed and we added either many counterfactuals (many counterfactuals version), a single counterfactual (single counterfactual version), or a reality statement (reality version) as a comment from the protagonist at the end of the story. In Experiment 1, we used the same reality statement in both many and single counterfactuals conditions. The stories are included in the Supplementary Appendix A.

Because we did not predict any difference between the stories we fixed their order (plane always first). We also fixed the order in which the versions of the stories were presented such that participants all have many/single version first in the plane story and reality version first in the fire story. It is common in counterfactual thinking experiments to present separate groups of participants with closely matched versions of the same story (e.g., Kahneman and Tversky, 1981; Roese and Olson, 1996). In our study we went a step beyond this, presenting individual participants with a pair of stories to ascertain whether participants made inferences based on counterfactuals when the counterfactual statements were highly salient.

In summary, the comparison between counterfactual and reality statements was tested in all participants, but half the participants compared many counterfactuals and reality and half compared a single counterfactual and reality.

This first study in our series was somewhat exploratory and has its limitations. We did not counterbalance the order of reality and counterfactual statements (the former always came first). This limitation is addressed in the subsequent studies. We also had a relatively small sample which we discuss further in the Section “General Discussion.”

#### Procedure

The experimenter greeted the participants and asked them to complete the consent form. Participants completed the experiment in a quiet room alone. After reading the pair of plane story versions (i.e., “many” and “reality,” or “single” and “reality”), participants were asked, “Although you cannot tell for sure from the reading materials, judging from what Michael/John said, who was closer to catching the flight?” Then they were asked “Please give any reasons why you think the protagonist who you chose (i.e., Michael or John) was closer to catching the flight?” Matched questions were used for the fire story, “Although you cannot tell for sure from the reading materials, judging from what Greg/Jack said, who might have been closer to remembering to send in the policy on time (i.e., the time the fire occurred was closer to the time that he forgot to send in the policy)?”, and “Please give any reasons why you think the protagonist who you choose (i.e., Greg or Jack) might have been closer to remembering to send in the policy on time?”. Participants were thanked and debriefed.

### Results and Discussion

Data for Experiment 1 are summarized in Table 1. First, we made a comparison between the many and single conditions. There was no difference in the pattern of choosing the counterfactual or reality versions of the stories between the many and single conditions: plane story, Fisher’s Exact Test (1, *N* = 42) *p* > 0.999, Cramer’s *V* = 0.06; fire story, Fisher’s Exact Test (1, *N* = 42) *p* = 0.697, Cramer’s *V* = 0.12.

**TABLE 1:** Number of participants inferring that the counterfactual version or reality version was closer in time.

		Version judged to be close	
		Counterfactual	Reality
Plane story	Single	17	4
	Many	16	5
Fire story	Single	16	5
	Many	18	3

Second, we explored whether participants were more likely to attribute temporal closeness to the protagonists making counterfactual statements, using Binomial tests to compare the responses in each condition. For the plane story participants were more likely to say that Michael (who made counterfactual statements) had missed the plane by a shorter time than John (who made a reality-based statement), many condition: *p* = 0.027, single condition *p* = 0.007; for the fire story, participants were more likely to say that Jack (who made counterfactual statements) might have been closer to remember to send in the policy than Greg (who made a reality-based statement), many condition *p* = 0.001, single condition *p* = 0.027.

To sum up, in Experiment 1 we found that the existence of counterfactual thoughts (compared to their absence) did lead participants to infer temporal closeness, but there was no difference in the pattern of choosing the counterfactual or reality versions of the stories between the many and single conditions. Therefore we found a difference in inferences about temporal distance based on whether there were (or were not) any counterfactual statements in the story. The results suggest the existence of counterfactual statements can lead participants to infer that events are closer than the absence of counterfactual thoughts. However, many counterfactuals did not lead participants to infer that events are closer than single counterfactual. These results are plausible because previous experiments did not explore the difference in the number of counterfactuals generated from closeness. Specifically, previous experiments (e.g., Meyers-Levy and Maheswaran, 1992; Roese and Olson, 1996 experiment 3) reported that temporal closeness (compared with temporal distance) was more likely to lead participants to engage in counterfactual thinking, rather than temporal closeness resulting in more counterfactual thoughts (compared to fewer). So in turn, it might be that the existence of counterfactual statements (compared to their absence) might prime judgments about temporal distance.

The alternative possibility for the lack of a difference between many and single counterfactuals might be that Experiment 1 always contrasted the many or single counterfactual statement with no counterfactual statements. Perhaps we are close to a ceiling effect when people compare any counterfactuals with none. In a follow up experiment we presented the counterfactual conditions separately to see if participants drew different inferences from them.

## Experiment 2: Closeness Follow-Up

In Experiment 1, we demonstrated that the existence of counterfactual thoughts led participants to infer that events are closer in time. However, we did not find a difference in inferring closeness based on reading many and single counterfactuals. As we mentioned above, one possibility is that the difference between many and single counterfactual statements may be hidden because of a near ceiling effect when comparing some counterfactuals to none. However, an alternative possibility is that there is no difference in inferring closeness based on reading many and single counterfactuals. When someone generates many counterfactuals perhaps they are exploring different counterfactual possibilities, but this does not necessarily indicate any greater “degree” of counterfactual thinking. Take the plane story as an example, the protagonist’s counterfactual statements in the many counterfactuals version of the story involve mental mutation of the departure time (e.g., “I should have left earlier”), the traffic (e.g., “If we thought about the traffic in advance, we would have not been blocked on the road”), and the ticket information (e.g., “If we booked earlier tickets, then we would have avoided this traffic jam”). On the other hand, the protagonist’s counterfactual statements in the single counterfactual version story only involve mental mutation of the departure time (e.g., “I should have left earlier”), which is only one aspect of the counterfactual events.

Therefore, in Experiment 2 we checked Experiment 1’s finding that there was no difference in inferring closeness based on reading many and single counterfactuals, by focusing on the difference between many and single counterfactual statements. In this study participants read a version of the story that had either many or single counterfactuals and judged the closeness of the past event. We hypothesized that knowing that a protagonist had generated many counterfactuals would result in participants inferring that the past event was closer in time compared to a protagonist who had generated a single counterfactual.

### Method

#### Participants

Eighty psychology students (63 women and 17 men) from a university in a city in the United Kingdom participated in Experiment 2 to gain course credits.

#### Design and Materials

All participants completed both the many and single statements conditions. Each participant read *plane* and *fire* counterfactual stories which were same as Experiment 1 (see Supplementary Appendix B). Each story had a “many counterfactuals” version and a “single counterfactual” version. We counterbalanced the order of the “many counterfactuals” and “single counterfactual” versions, and we fixed the story order of the plane story (always first) and fire story. Therefore, each participant read either the “single counterfactual” version of the plane story and the “many counterfactuals” version of the fire story, or the “many counterfactuals” version of the plane story and the “single counterfactual” version of the fire story. After reading each story, participants were required to judge when the flight left (or when the fire occurred), and then explain their reasons. Although participants explained their reasons, we have not analyzed them and do not report them in this paper.

#### Procedure

After reading the many or single counterfactuals version of the plane story (depending on their allocated condition), participants were asked “Although you cannot tell for sure from the reading material, judging from Michael’s description, when do you think the plane took off: (A) at the scheduled time, an hour before Michael arrived at the airport; (B) 5 min before Michael arrived at the airport, 55 min later than scheduled.” Then participants were asked, “Please give any reasons why you think this is when the flight took off before Michael arrived.” After reading single or many counterfactuals versions of the fire story (the complementary version to that read for the plane story), participants were asked “Although you cannot tell for sure from the reading material, judging from Greg’s description, when do you think the fire occurred: (A) 3 days after he forgot to send the policy document; (B) 6 months after he forgot to send the policy document.” Participants were then asked, “Please give any reasons why you think this is when the fire occurred.” After each participant completed the questionnaire, the researchers presented a debrief sheet to the participant.

### Results and Discussion

Data for Experiment 2 are summarized in Table 2. We compared the number of participants judging that the missed event was close or distant in time when they read many or single counterfactuals. For the plane story, a Chi Square test (with continuity correction) showed that there was no difference in the likelihood of inferring temporal closeness or distance, depending on whether participants read many or single counterfactuals, χ^2^(1, *N* = 80) = 0.22, *p* = 0.636, Cramer’s *V* = 0.08. For the fire story, a Chi Square test (with continuity correction) showed that there was no difference in the likelihood of inferring temporal closeness or distance, depending on whether participants read many or single counterfactuals, χ^2^ (1, *N* = 80) = 0.50, *p* = 0.482, Cramer’s *V* = 0.11.

**TABLE 2:** Number of participants inferring that the event was close or distant in time.

		Event judged to be	
		Close	Distant
Plane story	Single	15	25
	Many	12	28
Fire story	Single	28	12
	Many	24	16

We also explored whether participants were more likely to infer temporal closeness than temporal distance for the protagonists making counterfactual statements, using binomial tests to compare the responses in each story. Results here were mixed. For the plane story, participants were not more likely to say that the protagonist who made counterfactual statements had missed the plane by a shorter (i.e., 5 min before) than longer time (i.e., an hour before) when reading the single counterfactual version *p* = 0.154; but they were more likely to say that the protagonist who made counterfactual statements had missed the plane by a longer time when reading the many counterfactuals version *p* = 0.017. On the other hand, for the fire story, participants were more likely to say that the protagonist who made counterfactual statements might have been closer (than further) in time to remembering to send in the policy when reading the single counterfactual version, *p* = 0.017; but participants were not more likely to say that the protagonist who made many counterfactual statements was closer in time to remembering to send in the policy *p* = 0.268. It is also clear from the table that story (plane or fire) differed in the likelihood of participants reporting whether the event was close or distant in time. Counter to our expectations, in Experiment 2, participants did not consistently attribute closeness following reading counterfactual statements. We speculated that the way in which we had framed these questions may have affected these absolute responses (was the event close or far in time), that did not happen in Experiment 1 where the responses were relative (who was closer to the past event). For example, in the plane story when making the absolute judgment participants may bring in their background knowledge about events, such as planes typically taking off at the scheduled time. Some participants mentioned details like this in their justifications. “It’s very unlikely to change the scheduled time.”

To sum up, Experiment 2, as a check on Experiment 1, showed that participants did not infer greater temporal closeness when reading stories with many counterfactuals rather than a single counterfactual. In Experiment 1 our main question was concerned with whether people inferred closeness when the read counterfactuals compared to no counterfactuals, but we were also interested to see that we did not get a difference between many/single counterfactuals. Experiment 2 replicated this finding that there were no differences in the inferences made from stories with many counterfactuals rather than a single counterfactual. These results were counter to the prediction that many counterfactuals might be more salient than a single counterfactual. However, we note that there may still be a difference if many and single counterfactuals were presented in a pair of stories for direct contrast. It remains possible that the tendency to infer closeness from counterfactuals relies on the direct contrast between examples. With this caveat, we decided to explore whether people made inferences other than closeness on counterfactuals. This would give a firmer base for future research on when these inferences might be made.

## Experiment 3: Controllability

The functional theory of counterfactual thinking (Epstude and Roese, 2008) suggested that counterfactual thinking focusing on controllable events or actions is helpful for promoting future performance, because people can only make efforts to improve the controllable parts. Girotto et al. (1991) showed that participants thought counterfactually when they learned events were controllable. In Experiment 3 we explored if the reverse inference is true. Girotto et al. (1991) asked participants to generate counterfactual thoughts for one protagonist who was delayed by both controllable events (e.g., having a drink) and uncontrollable events (e.g., road blocked by a flock of sheep), finding that participants’ counterfactual thoughts mainly focused on protagonist’s controllable action. Based on this, we hypothesized that the existence of counterfactual thinking can lead participants to infer that the event was controllable. In the original experiment (Girotto et al., 1991), participants read that a protagonist was postponed by some controllable and uncontrollable events, and participants’ counterfactual thoughts mainly focused on the uncontrollable events. In Experiment 3, we explored participants’ inferences about controllability by presenting counterfactual statements or narrative statements to participants. We hypothesized that knowing a protagonist made statement(s) about counterfactuals (compared to a protagonist who made statement about reality) would be more likely to result in participants inferring that the past event was controllable.

### Method

#### Participants

Experiment 3 was an online experiment using Sona System. Forty-nine psychology students (40 women and 9 men) from a university in a city in the United Kingdom participated in Experiment 3 to gain course credits.

#### Design and Materials

There were two conditions in Experiment 3: a many counterfactuals condition where participants read about a character generating several counterfactual statements about the event, and a single counterfactual condition where participants read about a character generating just one counterfactual statement about the event. Each condition also read a second version of the story with a character who made a reality statement.

The original story (Girotto et al., 1991 experiment 1) was about a protagonist who was delayed by uncontrollable events (e.g., a flock of sheep in the middle of the road) and controllable decisions (e.g., own decision to have a drink). When he came home he found his wife had a heart attack and she was dying. The specific controllable and uncontrollable events in the story were concealed and we added either many counterfactuals (many counterfactuals version), a single counterfactual (single counterfactual version), or a reality statement (reality version) as a comment from the protagonist at the end of the story (see Supplementary Appendix C). Participants in the many condition read two versions of the story: one with many counterfactuals and one with a reality statement. Participants in the single condition read two versions of the story: one with a single counterfactual and one with a reality statement. We also counterbalanced the order of the versions (counterfactual or reality statements) between stories. In Experiment 3, we used same reality statement in both many and single counterfactuals conditions.

#### Procedure

Participants first read the pair of controllability story versions (i.e., “many” and “reality,” or “single” and “reality”). Participants were then asked, “Although you cannot tell for sure from the reading materials, judging from what Mr. Bianchi/Williams said, which person was delayed by his own decision to go to a bar?” Then they were asked “Please give any reasons why you think the protagonist who you chose (i.e., Mr. Bianchi/Williams) was delayed by his own decision to go to a bar whereas the other person was delayed by a flock of sheep in the road.” Finally, participants received online debrief information.

### Results and Discussion

Data for Experiment 3 are summarized in Table 3. First we made a comparison between the many and single conditions. There was no difference in the likelihood of inferring controllability between the many and single conditions, Fisher’s Exact Test (1, *N* = 49) *p* = 0.495, Cramer’s *V* = 0.19.

**TABLE 3:** Number of participants inferring that the event was controllable.

	Version judged to be controllable	
	Counterfactual	Reality
Single	25	2
Many	22	0

Second, we explored whether participants were more likely to attribute controllability to the protagonist who made counterfactual statements. In the many condition, a Binomial test (*p* < 0.001) showed that participants were significantly more likely to say that Mr. Bianchi (who made counterfactual statements) was delayed by his own decision to go to a bar than Mr. Williams (who made a reality statement). In the single group, a Binomial test (*p* < 0.001) showed that participants were significantly more likely to say that Mr. Bianchi (counterfactual statement) was delayed by his own decision to go to a bar than Mr. Williams (reality statement).

Overall, these results supported our hypothesis that the existence of counterfactual thinking can lead participants to infer controllability. These results indicated that there was no significant difference in inferring controllability based on different numbers of counterfactuals.

## Experiment 4: Exceptionality

In the above experiments, we have explored two types of inference participants made from reading counterfactual statements: inferences about closeness and about controllability. In Experiment 4, we look at a third type of inference, that is, inference about exceptionality. Based on Kahneman and Tversky (1981) who demonstrated that exceptional events can lead participants to think counterfactually, we explored whether reading counterfactual thoughts would in turn lead to inferences of exceptionality. The experimental paradigm that previous experiments (e.g., Kahneman and Tversky, 1981) used was to present participants with a story in which the protagonist underwent several exceptional and usual events, and then the protagonist did not obtain the expected outcome.

### Method

#### Participants

Experiment 4 was also an online experiment using Sona System. One-hundred-and-twenty psychology students (103 women and 17 men) from a university in a city in the United Kingdom participated in Experiment 4 to gain course credits.

#### Design and Materials

Design of Experiment 4 was similar to that of Experiment 1 and Experiment 3. There were also two conditions in Experiment 4: a many counterfactuals condition and a single counterfactual condition. Each participant read two counterfactual exceptionality stories which adapted from Kahneman and Tversky (1981). The original story was about a protagonist who chose an exceptional (or unexceptional) route, however, later he unfortunately suffered a traffic accident in the route he chose. We also wrote either many or single counterfactuals as a character’s own statements, accompanied with another story using reality statement as the protagonist’s own statement (see Supplementary Appendix D). Participants in the many condition read two versions of the story: one with many counterfactuals and one with a reality statement. Participants in the single condition read two versions of the story: one with a single counterfactual and one with a reality statement. We counterbalanced the order of the versions (counterfactual or reality statements) between stories. In Experiment 4, we used same reality statement in both many and single counterfactuals conditions.

#### Procedure

After reading both versions of story, participants were asked “Although you cannot tell for sure from the reading materials, judging from what Mr. Jackson/Jones’s wife said, which person was more likely to drive home by the exceptional route (rather than stick to the regular route that he used to drive)”. Participants were asked “Please give any reasons why you think this person (which you selected) was more likely to drive home by the exceptional route (rather than stick to the regular route that he used to drive).” Participants were thanked and debriefed online.

### Results and Discussion

Data for Experiment 4 are summarized in Table 4. First we made a comparison between the many and single conditions. A Chi Square test showed that there was no significant difference in inferring exceptionality between the many and single conditions, χ*^2^*(1, *N* = 120) = 1.63, *p* = 0.202, Cramer’s *V* = 0.14.

**TABLE 4:** Number of participants inferring that the event was exceptional.

	Version judged to be exceptional	
	Counterfactual	Reality
Single	36	20
Many	49	15

Second, we explored whether participants were more likely to attribute exceptionality to protagonist who made counterfactual statements. In the many condition, a Binomial test showed that participants were more likely to say that Mr. Jones (who made many counterfactuals) was more likely to drive home by the exceptional route than Mr. Jackson (who made a reality statement), *p* < 0.001. In the single condition, a Binomial test showed that participants were significantly more likely to say that Mr. Jones was more likely to drive home by the exceptional route than Mr. Jackson, *p* = 0.045.

Overall, these results supported our hypothesis that the existence of counterfactual thinking can lead to inferring exceptionality. These results indicated that there was no significant difference in inferring exceptionality based on different numbers of counterfactual statements.

## General Discussion

To understand how people make inferences based on knowing that someone made counterfactual statements, this paper explored the possible bidirectional relation between counterfactuals and counterfactual determinants We based our experiments on previous research demonstrating that closeness, controllability, and exceptionality are more likely to lead participants to have counterfactual thoughts than distance, uncontrollability, and normality (e.g., Kahneman and Tversky, 1981; Girotto et al., 1991; Meyers-Levy and Maheswaran, 1992; Roese and Olson, 1996). In four experiments we tested whether people infer that the event was more likely to be a near miss, controllable, and exceptional when they hear someone using counterfactual statements to describe an event. We found that the existence of counterfactual thoughts (compared to their absence) did lead participants to infer temporal closeness in Experiment 1. Experiment 2, as a check on Experiment 1, supported the claim that there was no difference in inferring closeness based on reading many and single counterfactuals. Experiment 3 demonstrated that the existence of counterfactual thinking can lead people to infer controllability. Experiment 4 demonstrated that the existence of counterfactual thinking can lead people to infer exceptionality. In general, this paper explores the function of counterfactual thinking that is conveying information from others’ counterfactuals. We found that the existence (but not the numbers) of counterfactual thoughts can lead people to infer that event is close, exceptional, and controllable, which suggests that the relation between closeness/controllability/exceptionality and counterfactual thinking is bidirectional. We note that Experiment 2 showed that the tendency to infer closeness was not always seen when participants had to make absolute judgments about stories with either single or many counterfactual statements. Having demonstrated that the tendency to make inferences extends beyond closeness to controllability and exceptionality, we highlight the need for future research to investigate the conditions under which these inferences are made, in particular, the influence of general knowledge and whether a contrast between different types of statements is important.

In summary, our results suggest that the relation between closeness/controllability/exceptionality and counterfactual thinking is bidirectional. We already knew that events that were closely missed, controllable, and exceptional lead to counterfactual thinking. We show that the reverse pattern is also true: when people hear someone using counterfactual statements to describe an event they infer that the event was more likely to be a near miss, controllable, or exceptional. Our findings advance our knowledge of counterfactual bidirectional relation in showing that as well as making counterfactual inferences based on facts about the real world, people also make interferences about the real world, based on counterfactual worlds.

Some readers may be surprised by our inclusion of controllability in this list of determinants with a bidirectional relationship with counterfactual thinking. We made this decision based on early research by Girotto et al. (1991). However, some researchers have questioned the relationship between controllability and counterfactual thinking (Girotto et al., 2007; see also Pighin et al., 2021). For example, Girotto et al. (2007) found that when participants read vignettes as readers, participants’ counterfactual thoughts did focus on the controllable events experienced by the protagonist. However, when participants were instead actors who actually experienced the events, their counterfactual thoughts focused primarily on the uncontrollable events. In our Experiment 3, presumably our participants were more like the readers in these studies. But it remains open for empirical research whether there would be any difference in the inferences drawn in real life if one heard counterfactual statements: is hearing a counterfactual statement more like being a reader, an actor, or an observer (see Pighin et al., 2021).

We interpret these results in the light of norm theory (Kahneman and Miller, 1986). A norm is general knowledge from and expectation of specific events, which is formed from many past experiences. Norm theory suggests that people assess that whether an event is normal or exceptional based on to what extent this event matches general knowledge and expectation. Norm theory further suggests that the more an event is consistent with general knowledge and expectation, the less likely it is that this event would lead to thinking counterfactually. In contrast, when an event deviates from general knowledge and expectation, people will evaluate this event to be exceptional. Therefore people will construct how the event could match the norm, and thinking counterfactually is how people restore the exceptional event to the norm (Kahneman and Miller, 1986). It is possible that controllable or near miss events make up a relatively small proportion of events in daily life. Therefore, the occurrence of controllable or near miss events is relatively exceptional. By using norm theory, we can understand why people make similar inferences about these three determinants. To conclude, the original research demonstrated that people generated counterfactual thoughts based on closeness, controllability, and exceptionality that deviate from norm. In turn, our results showed that reading counterfactuals can lead to inferring closeness, controllability, and exceptionality, which may be because people can tell from counterfactuals that the original event did not meet the norm. Counterfactual thinking can help to understand the norm, indicating how the original event was deviated from the norm. Therefore, the specific forms of norm deviation (i.e., closeness, controllability, and exceptionality) can then be inferred from counterfactuals.

### Non-preparatory Functions of Counterfactual Thinking: Conveying Information

According to the functional theory (Epstude and Roese, 2008), counterfactual thinking serves the preparatory function of leading to a behavioral intention to change, and finally the change in future behaviors. This paper suggests a non-preparatory function (i.e., the function of conveying information). On knowing that someone has made counterfactual statements, people can make inferences of closeness, controllability, and exceptionality. Combining previous research (on determinants leading to counterfactual thinking), we suggested that the relation between closeness/controllability/exceptionality and counterfactual thinking is bidirectional. That is, counterfactual thinking leads people to identify there are certain determinants of counterfactual thinking. Specifically, like our own counterfactual thoughts, others’ counterfactual thoughts can also help us to identify key determinants of the counterfactual. From reading and listening others’ counterfactual statements, people can infer that this character who thought counterfactually could have caught the flight if they arrived earlier. Therefore, counterfactual statements convey additional information to listeners, beyond the information explicitly given. The explicit information in the counterfactual statement might communicate causal information (e.g., “If I had left home earlier, I would have caught the flight.”), but the additional inferences communicate other details about the event that are not necessarily causal. It will be interesting in future work to see whether in real life conversations people always draw inferences about closeness, controllability, and exceptionality or just some of these inferences, and indeed, whether this is automatic. In sum, making a counterfactual statement conveys information about the event to the listener both directly and indirectly.

We acknowledge some important limitations to our studies. First, this paper does not consider an important counterfactual determinant, outcome valance, as an experimental variable. Research (Roese, 1994, 1997) has shown that people’s counterfactual thinking mostly arises after negative outcomes. All of our vignettes centered on negative events, because we had intuited that this fit better with the counterfactual statements. However, future research could explore whether people infer that the speaker of a counterfactual has experienced a more negative situation compared to one who simply describes reality^1^.

Second, in our experiments the counterfactual statements we used varied. In Experiments 1, 3, and 4, we used conditional if-then statements which made specific reference to the consequent outcome. However, in Experiment 2 we used a ‘should’ statement which leaves the consequent implicit. Similarly some of our counterfactuals referred directly to alternative courses of action, while some left this to be inferred (e.g., in Experiment 4 Mr. Jones’ wife says “If it were a Tuesday, he would have been working at home.”). In Experiment 3 we made reference to regret in the many counterfactuals condition (although the results did not differ from the single counterfactual condition which did not make such reference). There was also variation in the specific content involved – sometimes the many counterfactual statements might have seemed quite repetitive. These variations were not systematic and it would be useful for future work to explore the impact of the content of the counterfactual statement and in particular whether it directly refers to an emotion (regret or guilt).

Third, in our studies, our participants always read two stories. In Experiments 1, 3, and 4 they made a comparison between a counterfactual and a reality version of the same story. This may have drawn attention to the counterfactual statements making participants more likely to make an inference based on them. In Experiment 2 participants did not compare versions of the same story, but they did read two stories one with counterfactuals and one without. This may still have highlighted the key information. In everyday life we would rarely hear two statements about the same situation and instead would make inferences based on one person’s statement. Thus, our research paves the way for future research in which participants make inferences without the counterfactual information being (potentially) highlighted in the way it was here. Our claim remains that people do make inferences about determinants based on counterfactuals, but the extent to which they do this in everyday interactions remains to be seen.

Finally, our sample sizes in some studies were relatively small and we did not conduct *a priori* power calculations to determine our sample size. Subsequently, we did not conduct *post hoc* power calculations for studies 1, 3, or 4 as the data was binary and non-parametric. However, for Experiment 2 (parametric data), we had power of 0.88 to detect medium effect sizes (of 0.5) with our sample of 80 participants in a 2 × 2 design. Our sample sizes were somewhat justified by referring back to similar published studies: Meyers-Levy and Maheswaran (1992, Experiment 2) recruited 63 participants for two conditions.

## Conclusion

In four experiments we explored the inferences people make when they learn that counterfactual thinking has occurred. Compared with knowing someone made a statement about reality, knowing that someone made counterfactual statements was more likely to lead to inferring closeness, controllability, and exceptionality. Previous research showed that events that were closely missed, controllable, and exceptional lead to counterfactual thinking, and our experiments indicated that the reverse pattern is also true. Together, these results indicated that inferential relation between counterfactual thinking and its determinants was bidirectional, demonstrating that people can make inferences about the real world based on counterfactual worlds. To conclude, our paper provides a more comprehensive picture of the functions of counterfactual thinking. We suggest that one non-preparatory function of counterfactual thinking is that conveying information from others’ counterfactuals. From reading and listening others’ counterfactual thoughts, we can identify key determinants of the counterfactual.

## Data Availability Statement

The raw data supporting the conclusions of this article will be made available by the authors, without undue reservation.

## Ethics Statement

The studies involving human participants were reviewed and approved by the STEM Ethics Committee, University of Birmingham. The patients/participants provided their written informed consent to participate in this study.

## Author Contributions

YX conceived the main ideas of four experiments, collected the data, conducted the data analyses, and wrote the main draft of this manuscript. SB contributed to study conception, design, and analysis. Both authors contributed to the article and approved the submitted version.

## Conflict of Interest

The authors declare that the research was conducted in the absence of any commercial or financial relationships that could be construed as a potential conflict of interest.

## Publisher’s Note

All claims expressed in this article are solely those of the authors and do not necessarily represent those of their affiliated organizations, or those of the publisher, the editors and the reviewers. Any product that may be evaluated in this article, or claim that may be made by its manufacturer, is not guaranteed or endorsed by the publisher.

## Footnotes

1 We thank an reviewer for this suggestion.
