# Supplementary material for: The Bidirectional Relation Between Counterfactual Thinking and Closeness, Controllability, and Exceptionality
Source: Front Psychol. 2022 Mar 9;13:732870. doi: 10.3389/fpsyg.2022.732870 (PMC8959919; doi:10.3389/fpsyg.2022.732870)
Supplement: Supplementary file 1 [file Data_Sheet_1.docx]

***Appendices***

***Appendix A: Stories Used in Experiment 1***

*Plane story* used in experiment 1. Text in square brackets [ ] was added for the version of the story in experiment 1.

Please read the reading material and answer the following questions:

Michael [John] was 25 years old, single, and a management trainee at a national bank in downtown Toronto [London]. After his first few months on the job, he was required to participate in a training seminar out of town. This was an important seminar; after completing it he could be certified and assigned his first management position. His flight was scheduled to leave at 4PM from Pearson Airport in Toronto [Stansted Airport in London], so he left early from work to drive to the airport. Having been to the airport several times recently to pick up clients as part of his job, Michael [John] had settled on a particular route that he liked to take. He decided that today he would stick to this favoured route to the airport. However, Michael [John] was soon caught in a major traffic jam. While he was driving, the airline called him, said the flight may take off at the scheduled time but it may also be delayed a little due to aviation control reasons [(which means John might still have a chance to catch the plane)]. Michael [John] went to the airport, still hoping to catch the flight. He arrived an hour after the scheduled departure time. When he got to the ticket desk, he learned that his flight had indeed left.

*No counterfactuals version*: The assistant on the desk told him the flight had gone, and the assistant also told him how close to his arrival time it was. After John heard what the assistant said, John said: “The traffic to the airport was really bad, that made me late.”

*Fewer counterfactuals version*: The assistant on the desk told him the flight had gone, and the assistant also told him how close to his arrival time it was. After Michael heard what the assistant said, Michael said: “If Ihad left home earlier, Iwould have caught the flight.”

*More counterfactuals version*: After Michael heard what the assistant said, Michael said: “IfIhad left home earlier,Iwould have caught the flight. Why didn'tIcheck the traffic conditions in advance? If onlyIcaught the flight rather than missed it,Iwould have been at the destination now. IfIthought about the traffic in advance,Iwould have not been blocked on the road. IfIbooked earlier tickets, thenIwould have avoided this traffic jam.Ireally feel regret.”

*Fire story* used in experiment 1*.* Text in square brackets [ ] was added for the version of the story in experiment 1.

*Please read the following interview about home insurance, and answer the following questions:*

“BecauseI[Greg/Jack] lived in an apartment,I[Greg/Jack] never thought that property insurance was necessary. However, after speaking with a friend who sold insurance,I[Greg/Jack] realized the importance of such insurance. Thus,I[Greg/Jack] had my [his] friend write up a policy thatI[he] could examine, sign, and send in later that day. Due to my [his] busy schedule, however,I[Greg/Jack] forgot to send in the policy. This turned out to be a big mistake because later, a fire ravaged my [his] apartment.I[He] lost everything and had no cover.

*No counterfactuals version*: After that, Greg said: “I lost all my belongings in the fire andIwas not covered by any insurance.”

*Fewer counterfactuals version*: After that, Jack said: “IfIhad remembered to send in the policy,Iwould have had insurance cover now.”

*More counterfactuals version*: After that, Jack said: “IfIhad remembered to send in the policy,Iwould have had insurance cover now. Why didn'tIsend in the policy right afterIdecided to buy it? If onlyIsent in the policy rather than forgetting to do so,Iwould have reduced the losses the fire brought me. IfIhad made a note to remind me,Iwould have avoided forgetting to send in the policy. If onlyIset myself a reminder to send in the policy,Iwould have had insurance cover now.Ireally feel regret.”

***Appendix B: Stories Used in Experiment 2***

*Plane story* used in experiment 2.

Please read the reading material and answer the following questions:

Michael was 25 years old, single, and a management trainee at a national bank in downtown Toronto. After his first few months on the job, he was required to participate in a training seminar out of town. This was an important seminar; after completing it he could be certified and assigned his first management position. His flight was scheduled to leave at 4PM from Pearson Airport in Toronto, so he left early from work to drive to the airport. Having been to the airport several times recently to pick up clients as part of his job, Michael had settled on a particular route that he liked to take. He decided that today he would stick to this favoured route to the airport. However, Michael was soon caught in a major traffic jam. While he was driving, the airline called him, said the flight may take off at the scheduled time but it may also be delayed a little due to aviation control reasons. Michael went to the airport, still hoping to catch the flight. He arrived an hour after the scheduled departure time. When he got to the ticket desk, he learned that his flight had indeed left.

Fewer counterfactuals version: When he found he had missed the flight, he said: “I should have left earlier.”

More counterfactuals version: When he found he had missed the flight, he said: “I should have left earlier. Why didn'tIcheck the traffic conditions in advance? If onlyIcaught the flight rather than missed it,Iwould have been at the destination now. IfIthought about the traffic in advance,Iwould have not been blocked on the road. IfIbooked earlier tickets, thenIwould have avoided this traffic jam.Ireally feel regret.”

*Fire story* used inexperiment 2*.*

*Please read the following interview about home insurance which is written by a college student named Greg Sawyer:*

“BecauseIlived in an apartment,Inever thought that property insurance was necessary. However, after speaking with a friend who sold insurance,Irealized the importance of such insurance. Thus,Ihad my friend write up a policy thatIcould examine, sign, and send in later that day. Due to my busy schedule, however,Iforgot to send in the policy. This turned out to be a big mistake because later, a fire ravaged my apartment.Ilost everything and had no coverage.

Fewer counterfactuals version:Ishould have thought about insurance earlier.”

More counterfactuals version: Why didn'tIsend the policy sinceIdecided to? If onlyIsent that policy rather than forgetting to do so,Iwould have had enough money now. IfIremembered to subscribe to insurance information alerts,Iwould have avoided forgetting to send the insurance policy. If only I’d set myself a reminder to send off the policy,Iwould have obtained financial compensation from the insurance company.Ireally feel regret.”

***Appendix C: Stories Used in Experiment 3***

*Controllability story* used in experiment 3. Text in square brackets [ ] was added for the second version of the story in experiment 3.

*Please read the reading material and answer the following questions:*

A bank employee, Mr. Bianchi [Williams], who worked in an agency situated in a village near to the one where he lived with his wife. The day of the accident he was going home after work but his progress toward home was delayed by an event. When he arrived home, Mr. Bianchi [Williams] found his wife on the floor. He realized that she had had a heart attack and she was dying. He tried to help her, but his efforts were in vain.

*No counterfactuals version*: Mr. Williams very sadly said: “I feel so sad for my wife.”

*Fewer counterfactuals version*: Mr. Bianchi very sadly said: “IfIhad come home early,Imight have been able to save my wife.”

*More counterfactuals version*: Mr. Bianchi very sadly said: “IfIhad come home early,Imight have been able to save my wife. IfIhad come home earlier,Iwould have been able to see her one last time. IfIhad come home earlier,Icould have been able to take her to the hospital.Ifeel really regret.”

***Appendix D: Stories Used in Experiment 4***

*Exceptionality story* used in experiment 4. Text in square brackets [ ] was added for the second version of the story in experiment 4.

Mr. Jones [Jackson] was 47 years old, the father of three and a successful banking executive. His wife has been ill at home for several months. On the day of the accident, Mr. Jones [Jackson] left his office at the regular time. He sometimes left early to take care of home chores at his wife’s request, but this was not necessary on that day. Mr. Jones [Jackson] chose a route. The accident occurred at a major intersection. The light turned amber as Mr. Jones [Jackson] approached. Witnesses noted that he braked hard to stop at the crossing, although he could easily have gone through. His family recognized this as a common occurrence in Mr. Jones [Jackson]’ driving.As he began to cross after the light changed, a light truck charged into the intersection at top speed, and rammed Mr. Jones [Jackson]’ car from the left. Mr. Jones [Jackson] was killed instantly.

*No counterfactuals version*: Mr. Jackson’s wife said “I feel so sad for my husband”.

*Fewer counterfactuals version*: Mr. Jones’ wife said “If it were Tuesday, he would have been working at home”.

*More counterfactuals version*: The Jones’s wife said “If only Jones chose the other route. If he went the other way, he might have been able to avoid the accident. If he had gone the other way, he might not have died. Why he had to go that route.Ishould have been able to remind him to follow the traffic rule.Ifeel so regret”.
